# Optimal needle characteristics for classical inferior alveolar nerve block anesthesia: a systematic review

**DOI:** 10.1186/s13005-025-00481-1

**Published:** 2025-02-03

**Authors:** Mennat Allah Ashraf Abd-Elsabour, Ayat Gamal-AbdelNaser

**Affiliations:** 1 Pediatric and Community Dentistry Department, Faculty of Oral and Dental Medicine, Ahram Canadian University, Giza, Egypt; 2 Department of Oral Medicine and Periodontology, Faculty of Oral and Dental Medicine, Ahram Canadian University, Giza, Egypt; 3 Faculty of Oral and Dental Medicine, Ahram Canadian University, 4th Industrial Zone, Banks Complex, 6th of October City, Giza, Egypt

**Keywords:** Aspiration, Conventional inferior alveolar nerve block, Dental anesthesia, Local anesthesia, Mandibular anesthesia

## Abstract

**Objectives:**

Although frequently applied, inferior alveolar nerve block (IANB) is associated with high rate of complications, beside its unpredictable success; partly due to improper needle choice. Evidence of the ideal needle contradicts in literature. Therefore, this review aims to assess the effect of needle characteristics on the outcomes of IANB.

**Materials and methods:**

A systematic search was performed on MedLine via Pubmed, Cochrane Library, LILACS, Science Open, EBSCOhost, Scopus, Egyptian Knowledge Bank (EKB), and Google scholar; beside searching grey literature and hand searching. The review included randomized controlled trials comparing needles of difference in any of the characteristics (gauge, length, bevel, alloy) used for IANB regarding their effects on pain, success of anesthesia and aspiration. The authors ran the search, selected the eligible studies, assessed the risk of bias using RoB 1 and extracted the data of the finally included studies. All the steps were performed in duplicates.

**Results:**

The search yielded a total of 2,812 records. After de-duplication and excluding ineligible studies by title and abstract then by full text, the review included nine eligible studies. The compared needle interventions included: gauges (23G, 24G, 25G, 26G, 27G and 30G), lengths (12 mm, 25 mm, 32 mm, 35 mm, 42 mm), and internal diameters (0.265 mm, 0.215 mm, 0.3 mm and 0.4 mm). All studies had high risk of bias, adopted different assessment methods for the outcomes, and included participants with differences in baseline characteristics.

**Conclusions:**

The level of the available evidence introduced by primary studies hinder concluding the optimal needle characteristics; keeping the research question unanswered. However, within the limitations of the heterogenous studies, available data favors thinner needles for less pain during needle insertion; otherwise, data of the other outcomes was inconclusive.

**Clinical relevance:**

Thinner needles are favored for less pain during insertion; but standardized future studies are essentially needed for solid conclusions. A detailed standard protocol is, therefore, proposed.

**Supplementary Information:**

The online version contains supplementary material available at 10.1186/s13005-025-00481-1.

## Introduction

Local anesthesia designates the temporary loss of sensation in a particular part of the body through applying or injecting certain agent; while maintaining full consciousness. Profound local anesthesia prevents transmission of pain sensation during painful dental procedures. Thereby, it plays a huge role in relieving fear and anxiety, allowing for efficient and painless dental treatment, and promoting a positive dental attitude [1].

Inferior alveolar nerve block (IANB) represents the most commonly used technique for anesthetizing lingual and inferior alveolar nerves in patients requiring dental treatment of a mandibular posterior tooth [2, 3]. The conventional technique aims to deposit the local anesthetic solution in the pterygomandibular space, targeting the mandibular foramen [4]. But unfortunately, the success rates vary greatly; owing to technical defects, anatomic variations and the presence of acute infections [4, 5]. The technique encloses a myriad of variables; each of which can affect the success of the IANB. The list of variables includes the angle of needle insertion, the used anesthetic material, and the characteristics of the needle (length, gauge, internal diameter, bevel and type of alloy) [4–6]. For a successful technique, all these variables need to be standardized to ensure a perfect technique; minimizing the complications and technical errors of IANB [5, 7].

Previous systematic reviews have addressed the technique and angles of insertion [4], and the used anesthetic [5]. However, to the best of our knowledge, the needle characteristics remain an unsettled issue.

The choice of the needle for anesthetic injection determines many important parameters in the anesthetic technique, such as the perceived pain during injection, the deflection of the needle from the point of insertion, the flow rate of the anesthetic solution through the needle lumen, the ability of the needle to penetrate a blood vessel, the ability of the needle to aspirate blood with minimal force, the possibility of needle fracture during insertion, and -most importantly- the success of the anesthetic technique [8–12].

Needles differ in their gauge, length, bevel, and other features. The needle gauge is defined as the internal or external diameter of the needle; where the larger the gauge, the smaller the diameter [6, 11].

Needle characteristics were studied extensively in literature, with hugely conflicting results and conclusions [6, 13–16]. Some authors concluded that the usage of short needles with thin lumen would elicit less pain for the patients and less trauma for the adjacent tissues [13, 17, 18]. On the other hand, other authors -who would prefer the long thick needle- claim the opposite way round. Furthermore, it was argued that short thin needles do not allow for effective aspiration, where the needle tip would not touch bone in most of the cases; leading to failure of the anesthetic technique [6].

Likewise, while many studies suggested that the larger the gauge of the needle, the less the pain perceived during the injection, and the less the probability of vascular penetration [6, 13, 14]; other studies found no significant effect of needle gauge on either pain perception during injection or vascular penetration [15, 16, 19].

Based on these unresolved debates, the optimal needle characteristics remain extremely controversial, although IANB represents a basic procedure in daily dental practice. Thus, this review is performed aiming to conclude the optimal characteristics of IANB needle.

## Materials and methods

The review aims to assess the effect of different needle characteristics on the outcomes related to the IANB. In other words, the review addresses the following question: In patients receiving IANB anesthesia, do changes in needle characteristics (including gauge, length, bevel, and internal diameter) affect the outcomes of anesthesia?

The protocol of the review was registered on PROSPERO with number CRD42024563734. It has been available online since the 10th of July 2024.

### Eligibility criteria

The review exclusively included randomized controlled trials (RCT) for patients receiving IANB; regardless to the reason for receiving the anesthesia, whether children or adults. This represented the participants (P) component of the PICO of the review. The review was restricted to RCTs as they produce the highest level of clinical evidence and the least risk of bias. Therefore, their results are considered the most reliable upon drawing conclusions and recommendations for clinical practice [20].

For a study to be included, it should be concerned with comparing needles of different characteristics (any of gauge, length, bevel, or internal diameter). The different needle characteristics signified the intervention (I) and control (C) elements of the PICO of the review.

The review focuses on a group of outcomes (O of PICO); primarily, (i) Patients’ pain perception during injection, (ii) Efficiency of IANB assessed by patients’ loss of sensation after IANB administration, and (iii) Time elapsed from IANB introduction to onset of the anesthetic effect. Furthermore, the review is concerned with a secondary outcome; namely, vascular penetration (positive blood aspiration).

However, studies were excluded if they didn’t mention excluding patients receiving any medications that alter pain sensation; while assessing pain perception.

The search had no limitations regarding the time or language of publication.

According to the eligibility criteria, studies reporting different techniques of IANB anesthesia were out of the scope of the review. Similarly, studies using computer-guided techniques using Wand syringe and those of vibratory stimulation (Vibraject or Dentalvibe) were excluded because they used the same needle as that used in the comparator group following the conventional technique.

Moreover, studies reporting needle deflection were excluded as the outcome has no reliable or valid assessment method. Also, studies assessing the needle deformation -with no clinical outcomes- were excluded; as the laboratory results were not correlated to patient-related outcomes.

### Information sources

A systematic search was obtained through following a detailed search strategy (Appendix-A) on multiple electronic databases: MEDLINE via PubMed, Cochrane Library, LILACS, Science Open, EBSCOhost, Scopus, Egyptian Knowledge Bank (EKB), and Google Scholar. Moreover, grey literature was searched through Open Grey; and the reference lists of the included studies were also screened for eligible studies.

### Selection process

As clarified, the search strategy was run on the named databases by the two authors independently. Afterwards, the search results were screened by title and abstract. The eligible or suspectedly eligible records were then read in full text to ensure eligibility. Records of studies were managed using Mendeley (Version 1.17.10) reference manager software. All the afore-mentioned steps were performed by the two authors independently, followed by a meeting to check the consistency of decisions. Any disagreement in the decisions was resolved by discussion. When the full text of a record was not possibly retrieved, the reviewers contacted the authors, journal and/or publisher twice.

### Data collection process

After agreeing on the inclusion of studies, data of each of the included studies was extracted in a standardized form. Similarly, this step was performed in duplicates. When some important information was unclear in a certain record in the form of missing participants, unclear data about the assessment method or bias reduction measures, the corresponding author was contacted twice for clarification. Failure to receive a response after contact for one month led to excluding the relevant article.

### Data items

For each study, we reported the study design, participants’ number and age group (adults vs. pediatrics), the reason for receiving IANB, the types of intervention and control, the outcomes measured, and the results.

**Risk of bias (RoB)** was assessed independently with the help of (RevMan 5.3) version 1 of the Cochrane risk of bias assessment tool for randomized trials (RoB 1). The tool consists of seven domains: random sequence generation (selection bias), allocation concealment (selection bias), blinding of participants and personnel (performance bias, ) blinding of outcome assessors (detection bias), incomplete outcome data (attrition bias), selective reporting (reporting bias) and other potential biases. Selective reporting bias was checked by exploring the study protocol if published.

In each domain, the risk of bias was judged as “low”, “high” or “unclear” according to Cochrane handbook for systematic reviews [20]. The decision for the assessment in each domain was justified using verbatim quotes from the assessed study. Any conflicts in the decision between the authors were resolved by discussion.

### Effect measures and synthesis methods

The unit of analysis was the IANB injection (needle prick). Despite the broad scope of the review, meta-analysis was planned in case of including more than three studies measuring the same outcome using consistent measuring methods of specific treatments Furthermore, as the review is addressing multiple types of interventions (multiple gauges, lengths or internal diameters of needles), whenever three or more interventions were reported by multiple studies with consistency, transitivity and with no high risk of bias, network meta-analysis was planned [21]. Combining results was not meant to be restricted to a single large comparison; but rather heterogenous set of treatments were planned to be fractionated into narrower, more homogenous comparisons [22]. If quantitative analysis was not applicable, a qualitative summary was reported in a narrative way.

### Reporting bias

When the number of studies included in one outcome was more than 10, publication bias was planned to be tested by funnel plot.

### Certainty assessment

The level of evidence was rated -following the methods of Cochrane systematic reviews [23]- using the GRADE guidelines into high, moderate, low, and very low.

### Reporting guidelines

The review was performed and drafted following the PRISMA guidelines and checklist [24].

## Results

The current review focuses on the effect of needle characteristics (including gauge, length, bevel and internal diameter) on the outcomes related to the IANB.

### Study selection

The search yielded a total of 2,812 records (2,228 by searching databases and 584 from other sources). The records were then de-duplicated and screened for eligibility.

**Fig. 1:**
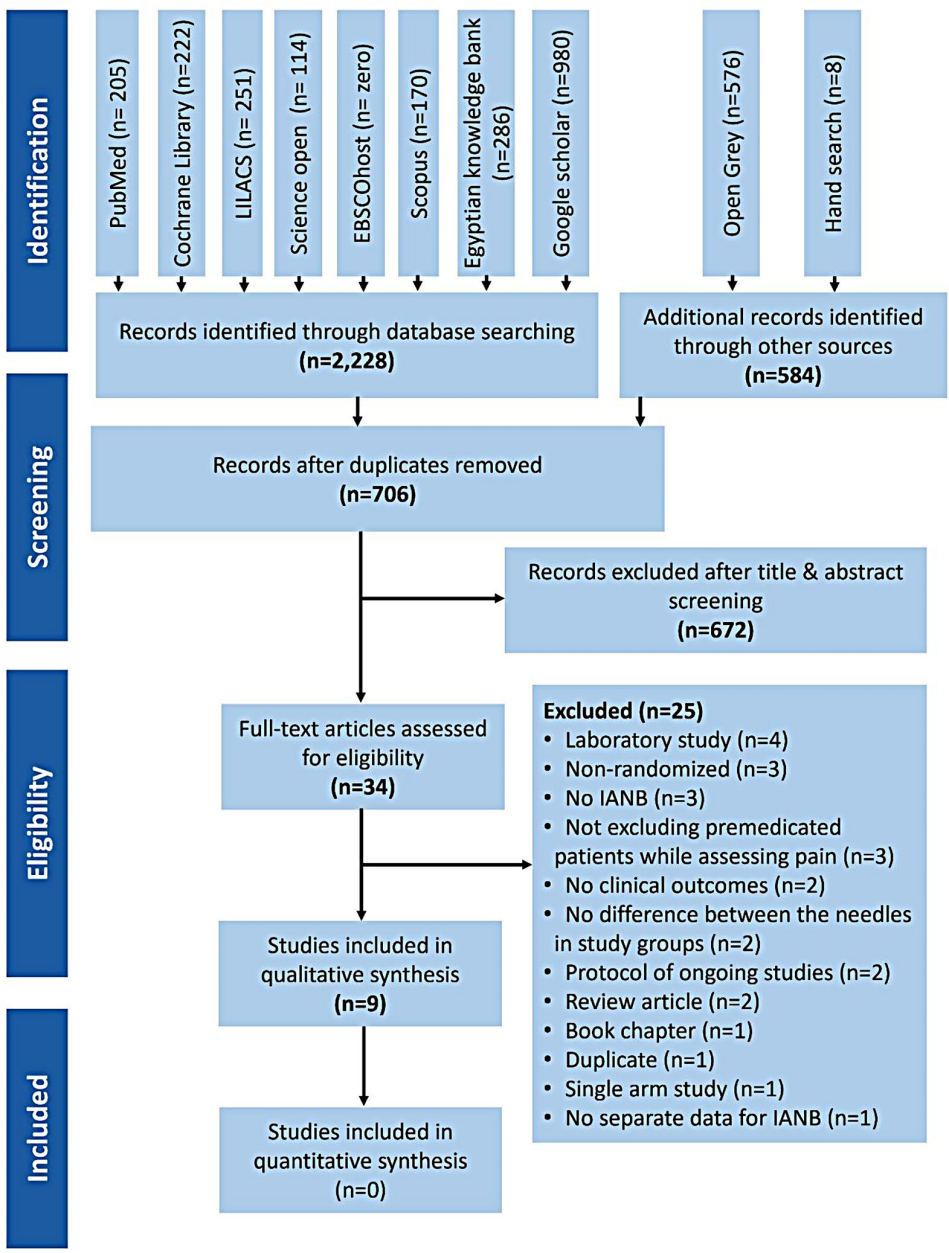
PRISMA flowchart of the steps of the review

Full texts of five records (four articles and one thesis) [25–29] were not retrievable even after contacting the author, or publisher twice; therefore, these records were excluded. One of the full texts screened [18] was concerned with all types of injections, and was later excluded after failure to receive a response regarding separate data of patients receiving IANB. Furthermore, two records of protocols were excluded after contacting the authors and ensuring the studies were still ongoing with no results yet available [30, 31] .The records excluded after full text screening were listed together with the reason of their exclusion (Appendix B). Finally, the review included nine eligible studies [6, 9, 13, 15–17, 32–34]. The number of records in each step throughout the process is clarified in Fig. 1.

**Table 1:** Characteristics of the included records

Study	Type of study	Participants/ injections	Reason of IANB	Intervention	Control	Outcomes	Results	Clinical conclusion
Al-Moraissi et al., 2021 [6]	RCT	212 Adults (106 injections per group)	Extraction of mandibular molars	27 gauge (32 mm long & 0.4 mm diameter) **Long + wide**	30 gauge (25 mm long & 0.3 mm diameter) **Short + thin**	Success by Dobbs scale (Mild, moderate, severe pain needing supplemental LA Or not)	**I** = 101/106 95.98% **C**= 66/106 ?? (62.2%) in text or 44/106 (41.5%) in the first figure or 45/106 (42.4%) in table *P* = 0.001	Long (**32 mm**) & wide (**27G**) needles yield lower pain & higher success of LA than short (**25 mm**) & thick (**30G**) ones.
						Pain during injection (VAS)	**Mean (SD)** **I** = 3.3 (1.22*)* **C** = 6.3 (1.3) *P* = 0.001	
Asokan et al., 2014 [17]	Cross-over RCT	30 Children (6–12 years old) But who received IANB (14 injections for I & 16 for C)	Pulp therapy or extraction (Not specified)	26 gauge	30 gauge	Pain using Taddio’s modified Behavioral Pain scale (1995) (crying, …)	**Mean (SD)** **I** = 2.71 (0.69) **C** = 2.21 (0.89) *P* = 0.104 **(NS)**	**30G** cause less pain than **26G**
Brownbill et al., 1987 [16]	RCT	138 Children (4–18 years old) Injections: (76 for I and 62 for C.)	Routine dental treatment (Not specified)	25 gauge, short needle	30 gauge, short needle	Success (by test of pain after 5 min.s by sharp explorer prick)	**I** = 36/76 (47%) **C** = 26/62 (42%)	**25G** has higher success than **30G** But **30G** have superior results in pain & aspiration than **25G**
						Pain (VAS)	**median** **I** = 13 **C** = 10	
						Aspiration by Watson & Colman score (5 levels)	**I** = 11/76 (14%) **C** = 11/62 (18%)	
Delgado-Molina et al., 2003 [15]	RCT	346 Adults Injections : (156 for I & 190 for C)	Extraction of the lower third molar	27Gx35mm with internal diameter of 0.265 mm	27Gx35mm with internal diameter of 0.215 mm	Aspiration as positive and negative	**I** = 15/156 (9.6%) **C** = 15/190 (7.9%)	**Big internal diameter**: better aspiration than **thinner of the same gauge.**
Fuller et al., 1979 [32]	RCT	6 dentists (each received 6 series of injections, each series consists of 3 injections of I_1_, I_2_ and C) = 108 (Injections: 36 /group)	Healthy volunteers	I_1_: 25G I_2_: 27G	30G	Pain during needle insertion (verbal into categories: none, mild, moderate, severe) then translated into numbers (1 to 7)	**Mean (SD)** **I**_**1**_ = 3.2 (1.4) **I**_**2**_ = 3.4 (1.4) **C** = 3 (1.4)	**Pain**: **27G > 25G > 30G**
Ghasemi et al., 2014 [13]	Cross-over RCT	40 children (5–8 years old) each receiving the 2 injections, (so, 80 injections: 40 per group)	Pulpotomy of second deciduous inferior molar teeth	27G Int. diameter 0.4 mm	30G Int. diameter 0.3 mm	Pain: Objective (by SEM score during injection) And Subjective (by FPS score after injection)	SEM Mean (SD) **I** = 1.5 (0.52) **C** = 1.17 (0.29) **Sig**. FPS **I =** 2.22 (1.02) **C** = 1.65 (0.76) **Sig**.	**30G** produce less pain than **27G**; Subj. and object.
						Success of injection (by pain and need for another injection)	100% in both groups	
Hussain et al., 2020 [9]	RCT	100 adults (50 injections per group)	Extraction of a lower tooth	23G needle on a 3 cc disposable syringe	27G needle on a metal dental syringe	Pain (numerical scale from 0–10)	**Mean (SD)** **I** = 4.50 (2.1) **C** = 3.86 (1.96) *P* = 0.167	**27G** produce less pain than **23G (**hypodermal**)**
Mazhar et al., 2020 [33]	RCT	100 Adult patients (50 injections each group)	For undergoing oral surgery of mandible	24G x 25.4 mm needle by 3 cc disposable hypodermic syringe	27G x42mm needle by conventional metallic dental syringe	Aspiration (+ ve or -ve)	**I** = 8/50 = 16% **C** = 15/50 = 30% **NS**	**27G** dental needle has better aspiration than **24G** of disposable hypodermal syringe
Stuepp et al., 2021 [34]	RCT	20 Adult participants; each receiving both injections (so, 40 injections: 20/group)	Not specified	12 mm long x 30G extra-short needle	35 mm long x 27G	LA onset By pressure & thermal testing at molars (within 12 min.: success)	**I**: 18/20 (90%) **C**: 17/20 (85%)	Extra-short **(12 mm) & 30G** has higher success & cause less pain than long **(35 mm) & 27G** BUT shorter duration of LA & more adverse events.
						LA duration (By pressure and thermal testing	**I**: 44.0 (19.55) min. **C**: 54.1 (13.7) min. *P* = 0.204	
						Pain during injection assessed (by a 100 mm VAS)	**I**: mean = **12.75 mm (13.3);** median = 8.5 mm **C**: mean = **15.9 mm (15.2);** median = 10.0 mm *P* = 0.398	
						Adverse events: (pain after 1 day of injection)	**I**: 8/20 (40%), **C**:6/20 (30%)	

C: Control; FPS: face pain rating scale; I: Intervention; IANB: Inferior alveolar nerve block: LA: local anesthesia; NS: non-significant; RCT: randomized clinical trial; SEM: sensory, eye and motor scale; SD: standard deviation; VAS: visual analogue scale

### Study characteristics

Nine studies [6, 9, 13, 15–17, 32–34] were included; six of them included adult patients; while the other three [13, 16, 17] targeted pediatric patients. All the included studies followed the RCT design; with two [13, 17] studies following the cross-over model of RCT. Some studies did not report the participants’ demographic data [9, 13, 15, 33]. The reasons for performing IANB differed between oral surgery and extraction [6, 9, 15, 33], pulpotomy of a lower deciduous molar [13] and injecting healthy volunteers [32]. On the other hand, some studies did not specify the reason [16, 17, 34]. Even for the studies that specified the reason for receiving IANB, the pulpal condition of the affected teeth was not always specified. The data of each of the included studies are detailed in Table 1.

The interventions compared in the included studies enclosed different needle characteristics: gauges (23G [9], 24G [33], 25G [16, 32], 26G [17], 27G [6, 9, 13, 32–34] and 30G [6, 13, 16, 17, 32, 34]), lengths (12 mm [34], 25 mm [6, 33], 32 mm [6], 35 mm [34], 42 mm [33]) and internal diameters (0.265 mm [15], 0.215 mm [15], 0.3 mm [6, 13] and 0.4 mm [6, 13]).

Likewise, the studies varied in the outcomes measured and the methods of assessing it. Pain was evaluated through visual analogue scale (VAS) [6, 16, 34], numerical scale [9], and Taddio’s modified behavioral pain scale [16, 35]. Other methods used for pain assessment included asking the patients to categorize pain verbally into none, mild, moderate or severe; then translating these categories into numbers (from 1 to 7) [32]. Lastly, combining subjective and objective methods for pain assessment was followed by one study [13]; which included pediatric patients. It used the face pain rating scale (FPS) as the subjective method; and the sound, eye, motor (SEM) scale as the objective method [36].

Other than pain, outcomes included anesthetic success which was evaluated using Dobbs scale [6, 37], testing pain after 5 min by a sharp explorer prick [16], pressure and thermal testing [34], together with the need for another injection [13]. Moreover, aspiration was assessed as positive or negative [15, 33]; and through using Watson & Colman score [16, 38]. Other outcomes were assessed by a solitary study [34]; namely duration of the anesthesia and the adverse effects.

### Risk of bias in studies

As the review only included RCTs, random sequence was generated in all the included studies rendering most of them [6, 9, 15, 32–34] of low risk of selection bias. Methods of randomization varied between computer-generation [6, 9, 34], coin tossing [15], alternation [33], and manual Table [32]. Nonetheless, the rest [13, 16, 17] did not provide information about the method used for sequence generation; therefore, they were assessed to have an unclear risk of bias.

Of all the included studies, only one [6] stated they concealed the allocation sequence using opaque envelopes; providing it low risk of selection bias. Otherwise, six studies did not report the allocation concealment; so, their risk of bias was rated as unclear. Lastly, two studies [33, 34] had high risk of bias; where allocation concealment was inapplicable in one of them due to allocating patients by alternation [33], while in the second study, randomization and allocation were made by the same surgeon who also performed the procedure [34].

As for the performance bias, only two studies reported blinding of the participants and operators [13, 16]; while another two studies did not mention any information about blinding causing their risk of performance bias to be assessed as unclear [15, 32]. In four studies, blinding of the operators was not applicable due to difference in the shape of the needles used in the two arms of the study; however, an unblinded operator raises a high risk of performance bias [6, 9, 33, 34]. Lastly, one study [17] did not perform blinding despite it was possible.

The risk of detection bias was assessed as low in three studies [6, 13, 34]; while it was high in one study [33] where the outcome assessor was not possibly blinded due to the used interventions and assessment method. Otherwise, the rest five studies had unclear risk of detection bias [9, 15–17, 32]; where the outcome assessor blinding was not mentioned.

Attrition bias was not probable in the included studies as the intervention was performed and assessed at the same appointment. Therefore, all the included studies had low risk of attrition bias; except for one study [6] in which some participants were unjustifiably excluded after randomization. On the other hand, selective reporting was not detected in any of the studies; so, they all had low risk of reporting bias.

Despite uncommon, all the included studies but one [34] had high risks of other biases in the form of discrepancies in statistical analysis [6, 33], not explaining the method of sample size estimation [15–17], lack of baseline characteristics reporting [9, 13, 15, 33] and using a questionable assessment method [32]. Substantial discrepancies in statistical analysis of one study [6] needed clarification through asking the author via an email; but no response was received for this point.

**Fig. 2:**
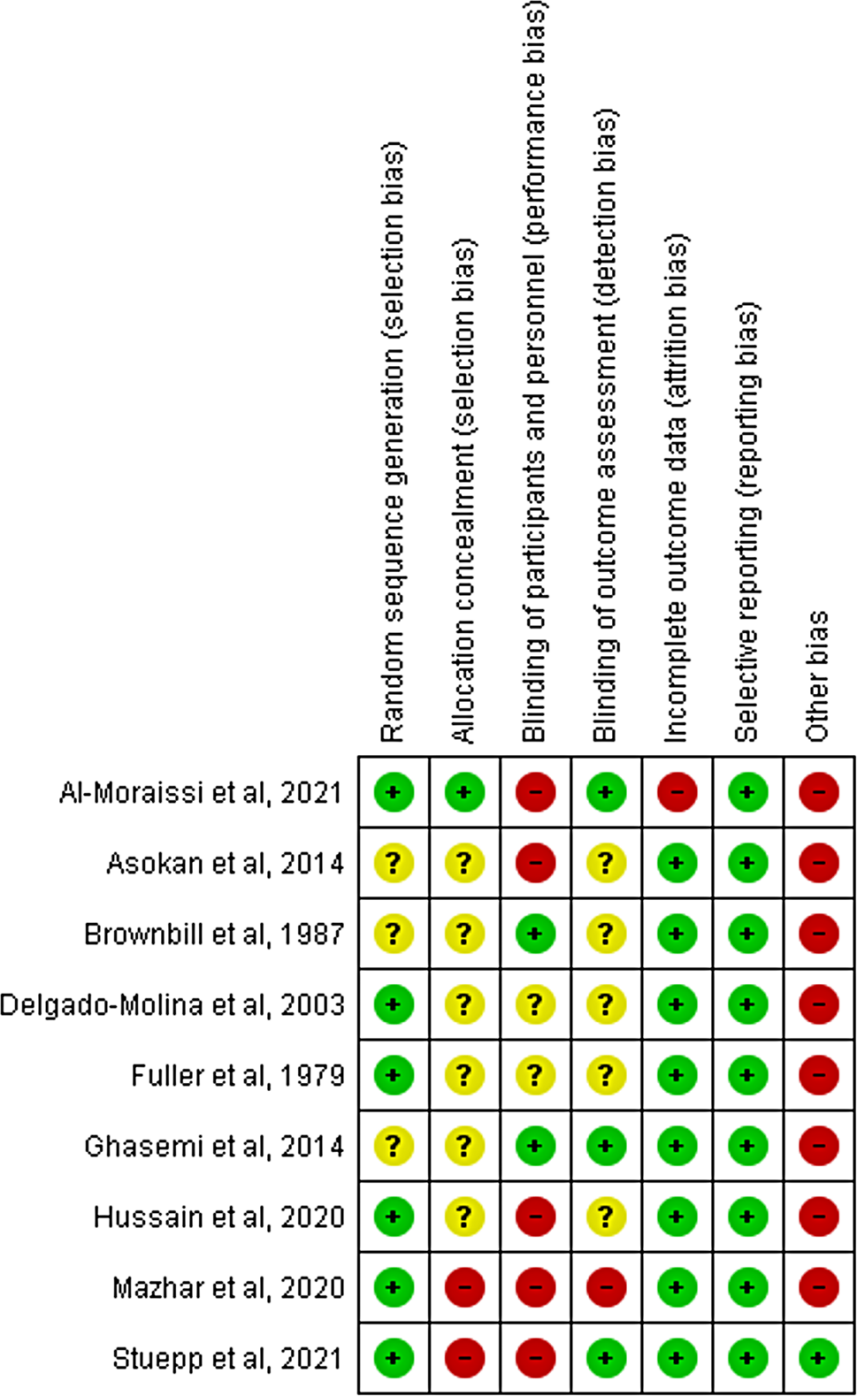
Results of risk of bias analysis of the included studies

In conclusion, all the included studies had high risk of bias through at least one of the assessed domains (Fig. 2). The details of risk of bias assessment and its justification are clarified in Appendix-C.

## Results of individual studies

The review addressed a group of outcomes:

1. **Patients’ pain perception during injection.** Seven of the nine included studies assessed pain during injection [6, 9, 13, 16, 17, 32, 34].

Regarding the gauge, 30G needles were compared to 25G [16], 26G [17] and 27G [6, 13, 34] separately. In only a single study [6], pain was found to be significantly higher with 30G needles when compared to 27G needles. In contrast, in all other studies, 30G needles were reported to elicit less pain when compared to 25G [16], 26G [17] and 27G [13, 34] ones. Similarly, when 25G, 27G and 30G needles were all compared together in one study, 30G needles caused the lowest pain; with the highest pain caused by 27G [32]. On the other hand, when 27G needles were compared to 23G ones, 27G needles provoked less pain when compared to ordinary 23G needles of disposable syringes [9]. Generally, larger gauge needles (smaller lumen) were found to be less painful than smaller (wider lumen) ones [9, 13, 16, 17, 32, 34].

Regarding the length, 25 mm needles caused more pain when compared to 32 mm ones [6]; while 12 mm needles (extra-short) were less painful than 35 mm ones [34].

From the point of view of internal diameter, one study reported 0.3 mm diameter caused more pain when compared to 0.4 mm needles [6]; while the results were totally contrasted by another study [13].

However, as stated earlier, each study used a different outcome assessment tool. Furthermore, some studies used more than one intervention at once; like using long and thin needles versus short and wide ones. Thereby, specifying the effect of length or gauge separately becomes impossible.

2. **Efficiency/success of IANB** was assessed by four studies [6, 13, 16, 34]. 100% success was reported in both 27G needles with an internal diameter of 0.4 mm and 30G needles with an internal diameter of 0.3 mm [13]. 25G needles showed slightly higher success rates (47%) when compared to 30G needles (42%) [16]. Also, 27G, 32 mm long needles with an internal diameter of 0.4 mm had higher success rates (95.98%) when compared to 30G, 25 mm long needles with an internal diameter of 0.3 mm (62.2%) [6]. Lastly, 30G, extra-short (12 mm) needles had a slightly higher success rate (90%) than 27G, 35 mm long needles (85%) [34]. The failure rates ranged between 0 and 58% in all studies.
3. **Time elapsed from IANB introduction to onset of the anesthetic effect** was not reported by any of the included studies in the form of minutes till onset; but rather as success or failure.
4. **Vascular penetration (positive blood aspiration)** was evaluated by three studies [15, 16, 33]. Higher aspiration rates were reported in 30G needles (18%) compared to 25G needles (14%) [16]. Also, needles of the metallic syringe of 27G and 42 mm length provided higher aspiration rates compared to disposable plastic syringes with 24G and 25 mm length [33]. On the other hand, expectedly, a larger internal diameter (0.265 mm) gave higher rates of aspiration (9.6%) when compared to a smaller diameter (0.215 mm) (7.9%) [15].

Other than the pre-specified outcomes, a solitary study [34] reported anesthesia delivered by 30G, extra-short (12 mm) needles lasted for a shorter duration and caused more adverse events than 27G, 35 mm long needles.

### Results of syntheses

The included studies suffered from substantial clinical and methodological heterogeneity; making meta-analyses impossible. As shown in Table 1, the studies differed hugely regarding study designs, age of the studied population, cause of receiving the injection, interventions tested, outcomes measured, and the methods of outcome assessment.

Pain assessed by VAS represented the only outcome that was evaluated by the same method in three studies [6, 16, 34]. Two of them, compared needles with different gauges and lengths at the same time [6, 34]; and the third [16] addressed pediatric patients only. Moreover, all the included studies suffered from an overall high risk of bias. Therefore, based on the extensive heterogeneity and high risk of bias, meta-analyses and network meta-analysis were out of the question.

### Certainty of evidence

In all the outcomes of the review, the eligible studies were limited in number, had considerable risk of bias and had heterogeneous inconsistent results. Therefore, the results concluded in this review are graded as being of very low quality of evidence. The summary of findings of each study is clarified in Table 1; while the grounds of grading of the evidence are discussed in appendix D.

## Discussion

Although conventional IANB is inevitable in everyday dentists’ practice, it is reported to cause the most frequent complications among all dental anesthetic techniques [39, 40]. These complications are attributed to a bunch of factors; including the choice of the needle. Literature includes contradicting evidence regarding the best needle characteristics to suit IANB; minimizing the complications and boosting the success rate [6, 13–16]. As the contradiction remains unresolved, this review was planned to assess the effect of different needle characteristics on the outcomes related to IANB; and to conclude practical recommendations accordingly.

The outcomes assessed in the review include pain perception during injection, efficiency (success) of IANB, and vascular penetration (positive blood aspiration).

Regarding pain perception during injection, the 30G needle (smaller lumen) proved to be superior (less painful) to 25G, 26G, and 27G [16, 17]. Generally when comparing different gauges, the larger gauge needle (smaller lumen) yielded less painful experience [9, 32, 34]. This finding can be attributed to the fact that the larger gauge needle (smaller lumen) requires less force to penetrate tissues and thus results in less pain during needle insertion [9, 13, 16, 17, 34].

In contrast, these findings seemed to be opposed by a single study [6]; which reported that the short 30G needle (smaller lumen) provoked more pain compared to the long 27G needle (wider lumen). This contradiction could be explained by the methodology of this study; where in the two arms of the study, the solution was injected within the same period of time (60 s).

According to Bernoulli’s principle, the narrower the lumen, the higher the velocity of the solution; given that the rate of flow of the solution is fixed [41]. Thus, by fixing the time needed for anesthetic injection and the volume of the injected anesthetic solution, the solution delivered through the smaller lumen needle (30G) will flow at a higher velocity towards the tissues. This rapid injections are believed to tear the tissues; causing immediate discomfort followed by persistent soreness for days after the anesthetic solution dissipates [42].

Being put together, as the results of all the studies may seem as if they contradict, they in fact complete the picture. During needle insertion, thinner needles were reported to cause less pain [9, 13, 16, 17, 34]; but during anesthetic deposition, they provoke more pain if the injection time was fixed [6]. However, this confusion and misconception of contradicting results arose as a result of inaccurate naming of the outcomes in the studies. The afore-cited studies assessed the pain of the process as a whole; without specifying the patient’s sensation during each step separately: needle insertion versus anesthetic deposition.

Shifting to the second primary outcome, when considering the success rate of the IANB, the included studies reported inconsistent results. The inconsistency could be justified by the considerable failure rate of IANB as a technique; especially in cases of pulpal inflammation [43–46]. Accordingly, the failure rates reported in the included studies (0–58%) are consistent with the overall range of failure rates of the technique reported in literature (7-77%) [43–46]. Therefore, the differences between success rates reported with the different needle gauges could be attributed merely to chance [5].

As the secondary outcome of the review, aspiration represents a recommended standard practice in IANB technique; aiming to avoid injecting the anesthetic solution into blood vessels. This step is considered crucial as intravascular injection may lead to remote or systemic complications; beside the decreased efficacy of the anesthesia [47]. The included studies showed different results. Surprisingly, larger gauge (smaller lumen) needles were reported to yield more frequent positive aspiration [16, 33]; except for a single study [15], where the needle with a wider internal gauge had a higher rate of positive aspiration. The results could be justified by the findings of a previous in-vitro study [48], where the 30G needle -which is the narrowest needle used in dental injections- was found to be able to aspirate blood. Therefore, the needle gauge seemed to have nothing to do with aspiration efficiency; where the narrowest lumen does not obscure aspiration. As a technique, IANB generally has high rates of positive aspiration owing to many factors other than the needle gauge; such as the anesthetic technique, the type of syringe, the experience of the operator, and -most importantly- the anatomic variations between patients [12, 49].

Lastly, the onset and duration of the anesthetic sensation were only reported by one of the included studies [34], which found that an ultra-short 30G needle resulted in delayed onset and shorter duration when compared with the standard 27G long needle. This could be attributed to the wide distance between the nerve site and the injection site when using the ultra-short needle. Therefore, the anesthetic solution takes more time to reach the nerve, and accordingly, more tissue absorption occurs, resulting in a smaller amount of the solution to reach the nerve; thus, a shorter anesthetic duration [34].

## Suggested guide for future research

To conclude the results, the addressed review question remained open for future research due to the huge heterogeneity of the studies methodologically and clinically. Therefore, and based on reviewing RCTs of the topic, we wish to recommend certain points upon designing future research.

We recommend conducting high-quality RCTs with adequate power calculation and detailed reporting of methods of randomization, and allocation concealment. Although blinding of the clinicians is not always possible when testing needles of different lengths, the participants can be blinded by placing a blindfold on their eyes during the procedure. Moreover, blinding of the outcome assessor is inevitable [50].

Considering the participants, it is advised to recruit participants who are receiving dental injections for the first time to avoid the effects of any previous dental experience on pain perception and anxiety; which may bias the results positively or negatively [51–53]. This recommendation is mostly applicable to studies targeting pediatric patients as it is uncommon for an adult to have no previous dental experience [54].

For the same reason of avoiding the effect of previous experience, the cross-over or split-mouth designs are prohibited; as the previous experience (of the first administered intervention) builds certain prospects about the second intervention. These expectations can easily bias the results of outcomes; mainly pain [51, 52].

As for the interventions, it is suggested that the authors should report a set of needle characteristics, such as the needle gauge (the internal and external diameters), the needle length, the type of alloy from which the needle is made, and any characteristics of the needle bevel. Furthermore, the trade name of the needle and the manufacturer should be named for reproducibility [20].

The studies are best designed to test one characteristic at a time so that the exact cause of the detected difference would be spotted; rather than testing different characteristics together like long and thin needles versus short needles with wider lumens. An alternative technique is proposed for addressing different categories of needle characteristics. It is advisable to utilize multifactorial RCT study design, in order to assess the effect of each feature separately.

Furthermore, all other factors (including the direction of the bevel) are advised to be standardized to avoid confounding the results [20].

Among the important factors to be standardized is the velocity of injection. It is recommended to use a fixed velocity of the solution during the injection through different needle gauges, instead of injecting in a fixed time period. The optimal injection rate of IANB has been debatable [42, 55, 56]; with a final conclusion that slower injection produces faster onset and higher efficiency of anesthesia.

The wider internal diameter of the needle has been claimed to facilitate the flow of the solution during injection with less need for pressure by the dentist. By doing so, it elicits less levels of pain. However, if the dentist uses the usual pressure, the flow rate will increase causing deposition of higher volume of solution in a shorter time; distending the tissues and causing more pain [19, 57].

Therefore, the optimal solution velocity was proposed to be (1 mL/minute); so, the deposition of the 1.8mL cartridge requires about 2 whole minutes. However, in everyday practice where dentists tend to inject the cartridge in no more than 20 s, recommending to deposit the cartridge in 60 s was found to be more pragmatic. One minute for the 1.8 mL would be slow enough to diffuse easily in the tissues without creating discomfort; and in case of accidental intravascular injection, would not cause serious reactions. In addition, slower injection over a sufficient period of time provided the anesthetic drug a chance to numb the injection area during the first couple of seconds, which make the rest of injection procedure more comfortable for the patient [58].

Nonetheless, this conclusion ignored the effect of the internal diameter of the needle on the actual velocity by which the solution is delivered to the tissues; owing to the notion of multiple studies that patients can rarely tell the difference between pain caused by different needle gauges [58]. However, in one of the included studies [6], the injection time span was fixed at one minute. Yet, a difference in pain perception in the two arms of the study was reported. This difference was attributed to the laws of physics stating that if injection time is fixed, the velocity of injecting a defined volume of fluid will be increased when using a narrower needle lumen, affecting the pain sensation of the patient [42]. Therefore, we recommend that it is the velocity of the deposited solution-not the time- that needs to be fixed for depositing the anesthetic solution with different needles.

Regarding the outcomes, studies are suggested to assess pain, success of anesthesia, duration of the anesthesia and if any adverse events at the site of injection. It is proposed to evaluate pain in both subjective and objective ways, especially in children who might not be able to properly assess their level of pain and express it themselves [59, 60]. These methods were applied by some of the included records [13, 30, 31].

Furthermore, pain is suggested to be assessed during each step separately: during needle insertion, during anesthetic solution deposition, and during needle withdrawal. This separation aims to avoid the confusion in interpreting the study results [61].

Moreover, the success of the IANB is advised to be evaluated as a function of loss of pulpal sensation after a certain period of time, through thermal or electrical pulp testing. As the pulp tissue is known to be the last to anesthetize, an anesthetized pulp confirms profound anesthesia [62]. This recommendation is best applied to a closed apex permanent tooth, while in young permanent and deciduous teeth, electrical and thermal pulp testing is not reliable and may yield false negative results [63].

As discussed, the review met a major limitation; namely the extensive clinical and methodological heterogeneity of the included studies. Besides, other limitations include the scarcity of RCTs with detailed reporting of important study characteristics causing a small number of finally included reports. Furthermore, the high risks of bias of the included studies impaired the certainty of evidence concluded by the review to very low quality of evidence.

## Conclusion

According to the available evidence, thinner needles cause less pain during insertion, but more pain during anesthetic deposition when injection time is fixed. The results of the effect of the needle on aspiration and anesthetic success were inconclusive. Thus, a group of recommendations was proposed for future studies to conclude the optimal standard characteristics of the needle used for IANB.

## Electronic supplementary material

Below is the link to the electronic supplementary material.

Supplementary Material 1

Supplementary Material 2

Supplementary Material 3

Supplementary Material 4

## Data Availability

No datasets were generated or analysed during the current study.
