# Supplementary material for: Optimal needle characteristics for classical inferior alveolar nerve block anesthesia: a systematic review
Source: Head Face Med. 2025 Feb 3;21:4. doi: 10.1186/s13005-025-00481-1 (PMC11789294; doi:10.1186/s13005-025-00481-1)
Supplement: Supplementary file 1 — Supplementary Material 1 [file 13005_2025_481_MOESM1_ESM.docx]

**Optimal Needle Characteristics for Classical Inferior Alveolar Nerve Block Anesthesia: A Systematic Review**

**Authors:** Mennat Allah Ashraf Abd-Elsabour^a^, Ayat Gamal-AbdelNaser^b*^

^a^Pediatric and Community Dentistry department, Faculty of Oral and Dental Medicine, Ahram Canadian University, Giza, Egypt.

^b^Department of Oral Medicine and Periodontology, Faculty of Oral and Dental Medicine, Ahram Canadian University, Giza, Egypt. Email: [ayat.gamal@acu.edu.eg](mailto:ayat.gamal@acu.edu.eg).

**Appendix -A- search strategy**

**#1. Inferior alveolar nerve block**

**#2. Inferior alveolar anesthesia**

**#3. IAN block**

**#4. (#1) OR (#2) OR (#3)**

**#5. Gauge**

**#6. 25 gauge needle**

**#7. 26 gauge**

**#8. 27 gauge**

**#9. 30 gauge**

**#10. Long needle**

**#11. Short needle**

**#12. Extra short needle**

**#13. Needle length**

**#14. Bevel**

**#15. Needle design**

**#16. Syringe**

**#17. Aspiration**

**#18. diameter**

**#19. (#5) OR (#6) OR (#7) OR (#8) OR (#9) OR (#10) OR (#11) OR (#12) OR (#13) OR (#14) OR (#15) OR (#16) OR (#17) OR (#18)**

**#20. (#4) AND (#19)**
