# Supplementary material for: Optimal needle characteristics for classical inferior alveolar nerve block anesthesia: a systematic review
Source: Head Face Med. 2025 Feb 3;21:4. doi: 10.1186/s13005-025-00481-1 (PMC11789294; doi:10.1186/s13005-025-00481-1)
Supplement: Supplementary file 2 — Supplementary Material 2 [file 13005_2025_481_MOESM2_ESM.docx]

**Optimal Needle Characteristics for Classical Inferior Alveolar Nerve Block Anesthesia: A Systematic Review**

**Authors:** Mennat Allah Ashraf Abd-Elsabour^a^, Ayat Gamal-AbdelNaser^b*^

^a^Pediatric and Community Dentistry department, Faculty of Oral and Dental Medicine, Ahram Canadian University, Giza, Egypt.

^b^Department of Oral Medicine and Periodontology, Faculty of Oral and Dental Medicine, Ahram Canadian University, Giza, Egypt. Email: [ayat.gamal@acu.edu.eg](mailto:ayat.gamal@acu.edu.eg).

**Appendix-B - Records Excluded by Full Text:**

|  | **Record** | **Reason for exclusion** |
| --- | --- | --- |
|  | (Aghahi et al. 2017) | No IANB |
|  | (Almendros Marqués et al. 2007) | No clinical outcomes |
|  | (Boonsiriseth et al. 2013) | No difference in needle characteristics in the study groups |
|  | (Canavan 2014) | Review article (Clinical feature) |
|  | (Cohen, Gravitz, and Knappe 1969) | Non-randomized, allocation according to the date of examination.  **Quote**: "For a period of two weeks, the dental officers each used a 27-gauge needle* with a lumen diameter of 0.008 inches. Then, for another two week period they used only 25-gauge needles having a lumen diameter of 0.010 inches." |
|  | (Cooley and Robison 1979) | Laboratory study |
|  | (Davidson 1989) | Laboratory study |
|  | (Delgado-Molina et al. 1999) | Not randomized |
|  | (Delgado-Molina et al. 2009) | No clinical outcomes |
|  | (E. Al-Moraissi, Al-Selwi, and Al-Zendani 2022) | Duplicate  Abstract of (E. A. Al-Moraissi, Al-Selwi, and Al-Zendani 2021) |
|  | (Flanagan et al. 2007) | No separate data for IANB. Excluded after contacting the author twice asking for the data with no response. |
|  | (IRCT20210720051947N1 2021) | Protocol of still ongoing study with no published results yet. |
|  | (Kotze and Labuschagne 2015) | No IANB |
|  | (Kour et al. 2017) | No IANB |
|  | (Kumar and George 2023) | Not randomized  **Quote:** "A random double-blinded crossover design was used" "Each patient received an injection with a 24-gauge needle during the first visit and during the second visit with the needle 26 gauge."  **Comment:** It is stated that all patients were apriori planned to receive the intervention in the first visit and the control in the second visit. So, this meant that it was not randomized; although labelled as random. |
|  | (Malamed 2011) | Review article |
|  | (McPherson et al. 2015) | did not exclude patients taking premedications affecting pain perception, while assessing pain |
|  | (NCT06242743 2024) | Protocol of still ongoing study with no published results yet. |
|  | (Ram, Hermida B, and Amir 2007) | did not exclude patients taking premedications affecting pain perception, while assessing pain |
|  | (Renton 2019) | Book chapter |
|  | (Robison et al. 1984) | Laboratory study |
|  | (Rood 1972) | Single arm study |
|  | (Sneharaj et al. 2024) | did not exclude patients taking premedications affecting pain perception, while assessing pain |
|  | (Steinkruger et al. 2006) | No difference in needle characteristics in the study groups |
|  | (Wittrock and Fischer 1968) | Laboratory study |

**References**:

Aghahi, Raha Habib et al. 2017. “Telescopic Dental Needles versus Conventional Dental Needles: Comparison of Pain and Anxiety in Adult Dental Patients of Kerman University of Medical Sciences—A Randomized Clinical Trial.” *Journal of Endodontics* 43(8): 1273–78. http://dx.doi.org/10.1016/j.joen.2017.03.027.

Al-Moraissi, EA, AM Al-Selwi, and EA Al-Zendani. 2022. “Needle Gauge and Inferior Alveolar Nerve Blocks.” *Dental Abstracts* 67(2): 112–13.

Al-Moraissi, Essam Ahmed, Abeer Mohammed Al-Selwi, and Elham Aziz Al-Zendani. 2021. “Do Length and Gauge of Dental Needle Affect Success in Performing an Inferior Alveolar Nerve Block during Extraction of Adult Mandibular Molars? A Prospective, Randomized Observer-Blind, Clinical Trial.” *Clinical Oral Investigations* 25(8): 4887–93.

Almendros Marqués, Nieves Almendros et al. 2007. “Comparison of Two Needle Models in Terms of Bevel Deformation during Truncal Block of the Inferior Alveolar Nerve.” *Medicina oral, patología oral y cirugía bucal* 12(4): 317–22.

Boonsiriseth, K., N. Sirintawat, K. Arunakul, and N. Wongsirichat. 2013. “Comparative Study of the Novel and Conventional Injection Approach for Inferior Alveolar Nerve Block.” *International Journal of Oral and Maxillofacial Surgery* 42(7): 852–56. http://dx.doi.org/10.1016/j.ijom.2012.11.017.

Canavan, Dermot. 2014. “Delivering Painless and Effective Inferior Alveolar Nerve Block Anaesthesia.” *Journal of the Irish Dental Association* 60(5): 230–32.

Cohen, M. B., L. A. Gravitz, and T. A. Knappe. 1969. “Twenty-Five versus Twenty-Seven-Gauge Needles.” *Journal of the American Dental Association (1939)* 78(6): 1312–14.

Cooley, Robert L., and Stephen F. Robison. 1979. “Comparative Evaluation of the 30-Gauge Dental Needle.” *Oral Surgery, Oral Medicine, Oral Pathology* 48(5): 400–404.

Davidson, Michael. 1989. “Bevel-Oriented Mandibular Injections: Needle Deflection Can Be Beneficial.” *General Dentistry* September-: 410–12.

Delgado-Molina, Esther, Susana Bueno-Lafuente, Leonardo Berini-Aytés, and Cosme Gay-Escoda. 1999. “Comparative Study of Different Syringes in Positive Aspiration during Inferior Alveolar Nerve Block.” *Oral Surgery, Oral Medicine, Oral Pathology, Oral Radiology, and Endodontics* 88(5): 557–60.

Delgado-Molina, Esther, Meritxell Tamarit-Borràs, Leonardo Berini-Aytés, and Cosme Gay-Escoda. 2009. “Comparative Study of Two Needle Models in Terms of Deflection during Inferior Alveolar Nerve Block.” *Medicina Oral, Patologia Oral y Cirugia Bucal* 14(9): 1–5.

Flanagan, Terry, Michael J. Wahl, Margaret M. Schmitt, and Jean A. Wahl. 2007. “Size Doesn’t Matter: Needle Gauge and Injection Pain.” *General Dentistry* 55(3): 216–17.

IRCT20210720051947N1. 2021. “Comparison of Two Needle Tip Designs ,Sebtoject and NOP on Pain Perception during Injection.”

Kotze, MJ, and W Labuschagne. 2015. “Evaluation of Two Different-Gauge Dental Needles for the Presence of Blood Following the Application of Local Anesthesia by Dental Students.” *South African Dental Journal* 70(1): 17–21. http://www.scielo.org.za/scielo.php?script=sci_arttext&pid=S0011-85162015000100005.

Kour, Gurpreet et al. 2017. “Insulin Syringe: A Gimmick in Pediatric Dentistry.” *International Journal of Clinical Pediatric Dentistry* 10(4): 319–23.

Kumar, Ashwin, and Melvin George. 2023. “COMPARISION OF 24 MM GAUGE AND 26 MM GAUGE FOR PAIN DURING INFERIOR ALVEOLAR NERVE BLOCK.” *Journal of Clinical Otorhinolaryngology, Head, and Neck Surgery* 27(2): 2848–55.

Malamed, Stanley F. 2011. “Is the Mandibular Nerve Block Passé?” *Journal of the American Dental Association* 142(September): 3S-7S. http://dx.doi.org/10.14219/jada.archive.2011.0340.

McPherson, Joanna Saenz, Sara A. Dixon, Richard Townsend, and Kraig S. Vandewalle. 2015. “Effect of Needle Design on Pain from Dental Local Anesthetic Injections.” *Anesthesia Progress* 62(1): 2–7.

NCT06242743. 2024. “Type of Needle Bevel on Pain Perception in Children During Inferior Alveolar Nerve Block Anesthesia.”

Ram, Diana, Laura Hermida B, and Erica Amir. 2007. “Reaction of Children to Dental Injection with 27- or 30-Gauge Needles.” *International Journal of Paediatric Dentistry* 17(5): 383–87.

Renton, Tara. 2019. “Optimal Local Anaesthesia for Dentistry.” *Primary dental journal* 7(4): 51–61.

Robison, S. F., R. B. Mayhew, R. D. Cowan, and R. J. Hawley. 1984. “Comparative Study of Deflection Characteristics and Fragility of 25-, 27-, and 30-Gauge Short Dental Needles.” *Journal of the American Dental Association (1939)* 109(6): 920–24. http://dx.doi.org/10.14219/jada.archive.1984.0246.

Rood, J. P. 1972. “Inferior Dental Nerve Block-Routine Aspiration and a Modified Technique.” *British Dental Journal* 132(February): 103–5.

Sneharaj, N., Akhilesh Sharma, Madhusudhan Kempaiah Siddaiah, and Priya Subramaniam. 2024. “Pain Perception in 4–6-Year-Old Children Following Intraoral Dental Injection with 26 and 31-Gauge Needles: A Randomized Controlled Trial.” *Journal of Dental Anesthesia and Pain Medicine* 24(2): 101.

Steinkruger, Geoffrey et al. 2006. “The Significance of Needle Bevel Orientation in Achieving a Successful Inferior Alveolar Nerve Block.” *Journal of the American Dental Association* 137(12): 1685–91. http://dx.doi.org/10.14219/jada.archive.2006.0114.

Wittrock, J. W., and W. E. Fischer. 1968. “The Aspiration of Blood through Small-Gauge Needles.” *Journal of the American Dental Association (1939)* 76(1): 79–81. http://dx.doi.org/10.14219/jada.archive.1968.0014.
