# Supplementary material for: Optimal needle characteristics for classical inferior alveolar nerve block anesthesia: a systematic review
Source: Head Face Med. 2025 Feb 3;21:4. doi: 10.1186/s13005-025-00481-1 (PMC11789294; doi:10.1186/s13005-025-00481-1)
Supplement: Supplementary file 3 — Supplementary Material 3 [file 13005_2025_481_MOESM3_ESM.docx]

**Optimal Needle Characteristics for Classical Inferior Alveolar Nerve Block Anesthesia: A Systematic Review**

**Authors:** Mennat Allah Ashraf Abd-Elsabour^a^, Ayat Gamal-AbdelNaser^b*^

^a^Pediatric and Community Dentistry department, Faculty of Oral and Dental Medicine, Ahram Canadian University, Giza, Egypt.

^b^Department of Oral Medicine and Periodontology, Faculty of Oral and Dental Medicine, Ahram Canadian University, Giza, Egypt. Email: [ayat.gamal@acu.edu.eg](mailto:ayat.gamal@acu.edu.eg).

**Appendix-C-Risk of bias assessment**

1. (Al-Moraissi, Al-Selwi, and Al-Zendani 2021)

| **Bias** | **Authors' judgement** | **Support for judgement** |
| --- | --- | --- |
| Random sequence generation (selection bias) | **Low risk** | **Quote:** " Random allocation sequence was generated using the computerized method." "Both generations of random sequence and allocation concealment were achieved prior to the beginning of the study by the first author (E.A)." |
| Allocation concealment  (selection bias) | **Low risk** | **Quote:** "Then, allocation concealment was done via an opaque sealed envelope to prevent selection bias in the recruitment stage. |
| Blinding of participants and personnel (performance bias) | **High risk** | **Quote:** "Because the type of intervention consisted of 2 types of the dental needles (short vs long or thinner vs thicker), both patients and investigator could not be blinding."  **Comment:** Although blinding was not applicable, the absence of blinding in this case brings along risk of performance bias. |
| Blinding of outcome  assessment (detection bias) | **Low risk** | **Quote:** "However, an assessment of outcomes was done by the blinded assessor (I.A)." |
| Incomplete outcome data  (attrition bias) | **High risk** | **Quote 1:** "Another twelve patients (6 patients in each group) were eliminated from the study because they received a second IANB injection."  **Comment 1:** Per-protocol analysis was performed rather than Intention-to-treat analysis. But the number of excluded participants was balanced in the 2 groups.  **Quote 2:** " Of two hundred and forty-one patients, twenty-nine patients were excluded because they did not meet the inclusion criteria. Two hundred twenty-four patients underwent randomization and allocation concealment and were included in the study. One hundred twelve patients received IANB using 27-gauge needles and one hundred and twelve patients received the 30-gauge needles. Another twelve patients (6 patients in each group) were eliminated from the study because they received a second IANB injection."  **Comment 2:** The number of patients in each step of the study does not lead mathematically to the final number of the included participants. 241 patients were recruited, 29 of which did not meet the eligibility criteria and were excluded. So, 241-29=212.  But, it was stated that the next step included 224 participants.  Afterwards, those 224 participants received the intervention and control injections; 112 per group. Yet, again it was stated that there were another 6 participants in each group who received the injections but were later excluded from analysis as they deviated from the protocol.  Furthermore, the number of these participants excluded in the analysis was stated as (6 patients in each group) within the text; but, was included in the flowchart as (5 cases were excluded) in each group. The number in text does not coincide with that in the figure. |
| Selective reporting (reporting bias) | **Low risk** |  |
| Other bias | **High risk** | The statistical analysis and included numbers of  participants show huge discrepancies:  **Quote 1**: "we required100 patients to receive IANB using 30-gauge needles, and 100 control patients receiving IANB using 27-gauge needles to challenge the null hypothesis". "Two hundred twenty-four patients underwent randomization and  allocation concealment and were included in the study."  **Comment 1:** Sample size calculation indicated the need for 200 participants, 100 per group. Yet, it was mentioned that randomization was performed to 224 participants without any justification of the added number of participants.  **Comment 2:** In the results of success of injection, 3 different results were reported for the same group (the control group of 30G needles): In the text, 66 out of 106 were reported; (with a wrong percentage of 41.5% instead of 62.2%);  while in figure 1, it stated 44 out of 106 (41.5%);  and in table 1 it added as 45 out of 106 (42.4%) |

1. (Asokan et al. 2014)

| **Bias** | **Authors' judgement** | **Support for judgement** |
| --- | --- | --- |
| Random sequence generation (selection bias) | **Unclear risk** | **Quote:** "The injection techniques were determined by random selection."  " A random crossover design was used. Every child acted as one’s own control, while receiving each treatment on the opposite side of the same arch."  **Comment:** It was stated that there was a random sequence but no information was included about the method of randomization. |
| Allocation concealment  (selection bias) | **Unclear risk** | **Comment:** Not mentioned. |
| Blinding of participants and personnel (performance bias) | **High risk** | **Quote:** "One assistant who was blinded to the type of injection and needle used, recorded the complete procedures in video recorder. All the injections were administered by the same operator who was not related the study."  **Comment:** The sentence clarified that the one shooting the videos was blinded and stated that the operator was not related to the study. So, the operator was not blinded.  Blinding of the operator was possible by obscuring the color codes on the needle caps.  There was no mention of participants' blinding. |
| Blinding of outcome  assessment (detection bias) | **Unclear risk** | **Quote:** "One assistant who was blinded to the type of injection and needle used, recorded the complete procedures in video recorder. All the injections were administered by the same operator who was not related the study."  **Comment:** There was no clarification of who assessed the outcomes; was it that assistant or one of the authors? and if they were blinded or not. |
| Incomplete outcome data  (attrition bias) | **Low risk** |  |
| Selective reporting (reporting bias) | **Low risk** |  |
| Other bias | **High risk** | **Comment:** Sample size calculation method was not explained; especially that they sub-grouped the 30 participants into 2 unequal groups based on their age (6-8y and 9-12y).  The cross over design was not properly applied; where the procedure was not necessarily the same in both times for each patient. Two patients received inferior alveolar nerve block versus infiltration. |

1. (Brownbill et al. 1987)

| **Bias** | **Authors' judgement** | **Support for judgement** |
| --- | --- | --- |
| Random sequence generation (selection bias) | **Unclear risk** | **Quote:** "Consecutive child patients (whose parents had given informed consent) requiring an inferior dental nerve block injection for treatment, were randomly assigned to the two needle gauge groups."  **Comment:** No statement of the method of randomization. |
| Allocation concealment  (selection bias) | **Unclear risk** | **Comment:** Not mentioned. |
| Blinding of participants and personnel (performance bias) | **Low risk** | **Quote**: "The distinguishing red and blue needle covers associated with 30- and 25-gauge needles, respectively, were replaced with yellow covers from 27-gauge needles when the syringes were prepared in the dispensary." |
| Blinding of outcome  assessment (detection bias) | **Unclear risk** | **Comment:** Not mentioned. |
| Incomplete outcome data  (attrition bias) | **Low risk** |  |
| Selective reporting (reporting bias) | **Low risk** |  |
| Other bias | **High risk** | **Comment:** Sample size calculation method was not explained; especially that the participants were unequally recruited to the 2 arms of the study (76 participants in the intervention group and 62 in the control group). |

1. (Delgado-Molina et al. 2003)

| **Bias** | **Authors' judgement** | **Support for judgement** |
| --- | --- | --- |
| Random sequence generation (selection bias) | **Low risk** | **Quote:** "Needle distribution was performed on a random basis by tossing a coin (heads: standard 27-gauge 35-mm needle with an internal gauge of 0.215 mm; tails: XL Monoprotect 27-gauge 35-mm needle with an internal gauge of 0.265 mm).” |
| Allocation concealment  (selection bias) | **Unclear risk** | Comment: Not mentioned. |
| Blinding of participants and personnel (performance bias) | **Unclear risk** | Comment: Not mentioned. |
| Blinding of outcome  assessment (detection bias) | **Unclear risk** | Comment: Not mentioned. |
| Incomplete outcome data  (attrition bias) | **Low risk** |  |
| Selective reporting (reporting bias) | **Low risk** |  |
| Other bias | **High risk** | **Comment 1:** Sample size calculation method was not explained; especially that the participants were unequally recruited to the 2 arms of the study (156 participants in the intervention group and 190 in the control group).  **Comment 2:** The technique of injection was not standardized in all participants. In both groups, some received injection by direct technique and others by indirect technique.  **Comment 3:** Baseline characteristics were not reported. |

1. (Fuller, Menke, and Meyers 1979)

| **Bias** | **Authors' judgement** | **Support for judgement** |
| --- | --- | --- |
| Random sequence generation (selection bias) | **Low risk** | **Quote:** "Participants were randomly placed within a sequence for a series of penetrations. The first participant began with series number one, the second began with series number two, and so forth, until each had received, in sequence, all six series  of penetrations."  **Comment:** The random sequence is shown in the article and seems to be manually performed. But the sequence was pre-planned before starting the study. |
| Allocation concealment  (selection bias) | **Unclear risk** | **Comment:** Not mentioned. |
| Blinding of participants and personnel (performance bias) | **Unclear risk** | **Quote:** "The participants in the study were never aware of which needle was used."  **Comment:** Blinding of the operators was not mentioned. |
| Blinding of outcome  assessment (detection bias) | **Unclear risk** | **Comment:** Not mentioned. |
| Incomplete outcome data  (attrition bias) | **Low risk** |  |
| Selective reporting (reporting bias) | **Low risk** |  |
| Other bias | **High risk** | **Quote:** "When the same verbal report given was noticed for two consecutive penetrations and when the latter penetration was reported of greater or less intensity, it was assigned the appropriate intermediate numeral on the continuum: 2,4, or 6.  For example, if both the first and second penetrations in a series elicited a mild sensation, 3, and the second injection was of greater intensity, it would be assigned a numerical value of 4."  **Comment:** The pain was reported verbally by the patient into categories as (none, mild, moderate and severe). Then, the outcome assessor translated it into number from zero to 7 based on the assessor's judgment of the participant. |

1. (Ghasemi, Rajaei, and Aghasizadeh 2014)

| **Bias** | **Authors' judgement** | **Support for judgement** |
| --- | --- | --- |
| Random sequence generation (selection bias) | **Unclear risk** | **Quote**: "This study was performed in a double-blind manner ".  "a random cross over design was used so that each child served as his or her own control; each patient was randomly assigned to receive the injection either with a 27- or 30-gauge needle for the second visit, while the injection with the other needle was administered during the third visit."  **Comment:** No statement of the method of randomization. |
| Allocation concealment  (selection bias) | **Unclear risk** | **Comment:** Not mentioned. |
| Blinding of participants and personnel (performance bias) | **Low risk** | **Quote:** "the pedodontist delivered randomly the topical anesthetic agents with different unknown needle"  "During the procedure, parents of children were not in operating room" |
| Blinding of outcome  assessment (detection bias) | **Low risk** | **Quote**: "a trained dental assistant also was blinded to the type of injection who was in charge of recording the behavioral parameters for every patient." |
| Incomplete outcome data  (attrition bias) | **Low risk** |  |
| Selective reporting (reporting bias) | **Low risk** |  |
| Other bias | **High risk** | **Comment:** Baseline characteristics are not reported. |

1. (Hussain et al. 2020)

| **Bias** | **Authors' judgement** | **Support for judgement** |
| --- | --- | --- |
| Random sequence generation (selection bias) | **Low risk** | **Quote:** "Each group had 50 participants which were randomly allocated using computer generated random tables." |
| Allocation concealment  (selection bias) | **Unclear risk** | **Comment:** Not mentioned |
| Blinding of participants and personnel (performance bias) | **High risk** | **Comment:** Not applicable due to difference in the shape of the syringe. But, risk of performance bias exists with absence of blinding. |
| Blinding of outcome  assessment (detection bias) | **Unclear risk** | **Comment:** Not mentioned |
| Incomplete outcome data  (attrition bias) | **Low risk** |  |
| Selective reporting (reporting bias) | **Low risk** |  |
| Other bias | **High risk** | **Comment:** Baseline characteristics are not reported. |

1. (Mazhar et al. 2020)

| **Bias** | **Authors' judgement** | **Support for judgement** |
| --- | --- | --- |
| Random sequence generation (selection bias) | **Low risk** | **Quote:** "For assigning the first patient to one of the groups, coin-toss method was used and was included in Group A. Every alternate patient was then included in group B (3cc disposable hypodermic plastic syringe)." |
| Allocation concealment  (selection bias) | **High risk** | **Comment:** Not applicable as they were allocated by alternation. |
| Blinding of participants and personnel (performance bias) | **High risk** | **Comment:** Not applicable due to difference in the shape of the syringe, yet unblinding of the operator may present performance bias in this case. |
| Blinding of outcome  assessment (detection bias) | **High risk** | **Comment:** Not applicable as the aspiration was assessed by looking at the syringe. With the difference in the shape of syringes, blinding is not possible. But, the assessment requires the assessor's judgment of the score. So, there is a risk of bias. |
| Incomplete outcome data  (attrition bias) | **Low risk** |  |
| Selective reporting (reporting bias) | **Low risk** |  |
| Other bias | **High risk** | **Quote 1**. ".....The sample size was calculated to be 81." "Among all the patients presenting ........, 100 patients requiring administration of inferior alveolar nerve block were selected"  **Comment 1:** Sample size calculation indicated the need for 81 participants; Yet, it was mentioned that 100 patients were included without any justification of the added number of participants.  **Comment 2:** Baseline characteristics are not reported.  **Comment 3:** The results were reported in absolute counts and in percentage. The percentage was calculated as if the group included 100 patients; while it only included 50 patients. |

1. (Stuepp et al. 2021)

| **Bias** | **Authors' judgement** | **Support for judgement** |
| --- | --- | --- |
| Random sequence generation (selection bias) | **Low risk** | **Quote:** "This is a randomized, split-mouth, double-blinded, noninferiority trial."  "The IANB technique that was to be performed on the right side was chosen using a randomization software program (Advanced Number Generator), whereas the other technique was performed on the contralateral side." |
| Allocation concealment  (selection bias) | **High risk** | **Quote:** "Allocation and randomization of participants were performed by the surgeon on duty, who also coded and registered any information regarding the anesthetic procedures  that were performed." |
| Blinding of participants and personnel (performance bias) | **High risk** | **Quote:** "Importantly, although the surgeon on duty was not blinded to the procedures, both participants and the outcome assessor (L.G.C.F.) were unaware of which technique was applied." "To warrant adequate blinding, the participants wore a pair of large glasses with opaque lenses which covered their whole field of view; also, any records of techniques applied were coded, and therefore they were accessible only to the surgeon on duty until the end of the study."  **Comment:** The participants were blinded; while blinding of the operator was not possible due to difference in the lengths of the needles. However, risk of performance bias exists due to unblinded operators. |
| Blinding of outcome  assessment (detection bias) | **Low risk** | **Quote:** "both participants and the outcome assessor (L.G.C.F.) were unaware of which technique was applied. In addition, to avoid bias from the outcome assessor resulting from identification of technique applied, local hemostasis was performed in the area of needle injection to avoid the presence of bleeding points." |
| Incomplete outcome data  (attrition bias) | **Low risk** |  |
| Selective reporting (reporting bias) | **Low risk** |  |
| Other bias | **Low risk** |  |

**References:**

Al-Moraissi, Essam Ahmed, Abeer Mohammed Al-Selwi, and Elham Aziz Al-Zendani. 2021. “Do Length and Gauge of Dental Needle Affect Success in Performing an Inferior Alveolar Nerve Block during Extraction of Adult Mandibular Molars? A Prospective, Randomized Observer-Blind, Clinical Trial.” *Clinical Oral Investigations* 25(8): 4887–93.

Asokan, Alexander et al. 2014. “A Pain Perception Comparison of Intraoral Dental Anesthesia with 26 and 30 Gauge Needles in 6-12-Year-Old Children.” *Journal of Pediatric Dentistry* 2(2): 56.

Brownbill, John W., Paul O. Walker, Bradley D. Bourcy, and Kathleen M. Keenan. 1987. “Comparison of Inferior Denial Nerve Block Injections in Child Patients Using 30-Gauge and 25-Gauge Short Needles.” *Anesthesia Progress* 34(6): 215–19.

Delgado-Molina, Esther, Meritxell Tamarit-Borrás, Leonardo Berini-Aytés, and Cosme Gay-Escoda. 2003. “Evaluation and Comparison of 2 Needle Models in Terms of Blood Aspiration during Truncal Block of the Inferior Alveolar Nerve.” *Journal of Oral and Maxillofacial Surgery* 61(9): 1011–15.

Fuller, N. P., R. A. Menke, and W. J. Meyers. 1979. “Perception of Pain to Three Different Intraoral Penetrations of Needles.” *Journal of the American Dental Association (1939)* 99(5): 822–24.

Ghasemi, Davoud, Sara Rajaei, and Ershad Aghasizadeh. 2014. “Comparison of Inferior Dental Nerve Block Injections in Child Patients Using 30-Gauge and 27-Gauge Short Needles Department of Pedodontics , Dental Faculty of Islamic Azad University of Khorasgan , Isfahan.” *JDMT* 3(2): 71–76.

Hussain, Noor UI Sabah et al. 2020. “COMPARISON OF PAIN PERCEIVED BY PATIENTS UNDERGOING INTRA ORAL LOCAL ANESTHESIA USING DIFFERENT NEEDLE GAUGES.” *Pak Armed forces Med J* 70(6): 1702–6.

Mazhar, Bushra et al. 2020. “COMPARISON OF POSITIVE ASPIRATION DURING INFERIOR ALVEOLAR NERVE BLOCK USING CONVENTIONAL METALLIC DENTAL SYRINGE VS. DISPOSABLE PLASTIC SYRINGE.” *Pak Armed forces Med J* 70(5): 1539–43.

Stuepp, Rúbia Teodoro et al. 2021. “Anesthetic Efficacy of an Alternative Inferior Alveolar Nerve Block Technique Using an Extra-Short Needle: A Double-Blind Randomized Non-Inferiority Trial.” *Journal of Oral and Maxillofacial Surgery* 79(5): 1025.e1-1025.e8.
