# Supplementary material for: Optimal needle characteristics for classical inferior alveolar nerve block anesthesia: a systematic review
Source: Head Face Med. 2025 Feb 3;21:4. doi: 10.1186/s13005-025-00481-1 (PMC11789294; doi:10.1186/s13005-025-00481-1)
Supplement: Supplementary file 4 — Supplementary Material 4 [file 13005_2025_481_MOESM4_ESM.docx]

**Optimal Needle Characteristics for Classical Inferior Alveolar Nerve Block Anesthesia: A Systematic Review**

**Authors:** Mennat Allah Ashraf Abd-Elsabour^a^, Ayat Gamal-AbdelNaser^b*^

^a^Pediatric and Community Dentistry department, Faculty of Oral and Dental Medicine, Ahram Canadian University, Giza, Egypt.

^b^Department of Oral Medicine and Periodontology, Faculty of Oral and Dental Medicine, Ahram Canadian University, Giza, Egypt. Email: [ayat.gamal@acu.edu.eg](mailto:ayat.gamal@acu.edu.eg).

**Appendix-D- Certainty of evidence**

**Certainty of evidence** of each outcome of the review is generally assessed through the GRADE approach. It is used to evaluate the confidence in the pooled estimates of the effect. Due to the impossibility to obtain a pooled estimate for the effect of any of the tested interventions, grading of evidence was assessed for the results of the individual studies separately:

| **Outcomes** | **Study** | **Quality of evidence** | **Justification** |
| --- | --- | --- | --- |
| **Pain** | **Al-Moraissi et al., 2021** [1] | ⊕⊝⊝⊝very low | Downgraded by one level for risk of bias  and two levels for imprecision due to inconsistencies in the statistics. |
|  | **Asokan et al., 2014** [2] | ⊕⊝⊝⊝very low | Downgraded by one level for risk of bias  and two levels for imprecision due to small sample size  and unequal groups. |
|  | **Brownbill et al., 1987** [3] | ⊕⊝⊝⊝very low | Downgraded by one level for risk of bias  and two levels for imprecision due to:  unjustified unequal grouping  and only stating the median with no variance. |
|  | **Fuller et al., 1979** [4] | ⊕⊝⊝⊝very low | Downgraded by one level for risk of bias  and two levels for imprecision due to unreliable assessment method. |
|  | **Ghasemi et al., 2014** [5] | ⊕⊕⊝⊝low | Downgraded by one level for risk of bias  and one level for imprecision due to small sample size |
|  | **Hussain et al., 2020** [6] | ⊕⊕⊝⊝low | Downgraded by one level for risk of bias  and one level for imprecision due to insignificant results. |
|  | **Stuepp et al., 2021** [7] | ⊕⊝⊝⊝very low | Downgraded by one level for risk of bias  and two levels for imprecision due to:  small sample size  and insignificant results |
|  |  |  |  |
| **Success of IANB** | **Al-Moraissi et al., 2021** [1] | ⊕⊝⊝⊝very low | Downgraded by one level for risk of bias  and two levels for imprecision due to inconsistencies in the statistics. |
|  | **Brownbill et al., 1987** [3] | ⊕⊕⊝⊝low | Downgraded by one level for risk of bias  and one level for imprecision due to:  unjustified unequal grouping |
|  | **Ghasemi et al., 2014** [5] | ⊕⊕⊝⊝low | Downgraded by one level for risk of bias  and one level for imprecision due to small sample size |
|  | **Stuepp et al., 2021** [7] | ⊕⊝⊝⊝very low | Downgraded by one level for risk of bias  and two levels for imprecision due to:  small sample size  and insignificant results |
|  |  |  |  |
| **Aspiration** | **Brownbill et al., 1987** [3] | ⊕⊕⊝⊝low | Downgraded by one level for risk of bias  and one level for imprecision due to:  unjustified unequal grouping |
|  | **Delgado-Molina et al., 2003** [8] | ⊕⊝⊝⊝very low | Downgraded by one level for risk of bias  and two levels for imprecision due to small sample size  and unstandardized methods in the 2 arms of the study. |
|  | **Mazhar et al., 2020** [9] | ⊕⊝⊝⊝very low | Downgraded by one level for risk of bias  and two levels for imprecision due to inconsistencies in the statistics. |

References:

1. Al-Moraissi EA, Al-Selwi AM, Al-Zendani EA (2021) Do length and gauge of dental needle affect success in performing an inferior alveolar nerve block during extraction of adult mandibular molars? A prospective, randomized observer-blind, clinical trial. Clin Oral Investig 25:4887–4893. https://doi.org/10.1007/s00784-021-03796-w

2. Asokan A, Rao A, Mohan G, et al (2014) A pain perception comparison of intraoral dental anesthesia with 26 and 30 gauge needles in 6-12-year-old children. J Pediatr Dent 2:56. https://doi.org/10.4103/2321-6646.137690

3. Brownbill JW, Walker PO, Bourcy BD, Keenan KM (1987) Comparison of inferior denial nerve block injections in child patients using 30-gauge and 25-gauge short needles. Anesth Prog 34:215–219

4. Fuller NP, Menke RA, Meyers WJ (1979) Perception of pain to three different intraoral penetrations of needles. J Am Dent Assoc 99:822–824. https://doi.org/10.14219/jada.archive.1979.0384

5. Ghasemi D, Rajaei S, Aghasizadeh E (2014) Comparison of Inferior Dental Nerve Block Injections in Child Patients Using 30-Gauge and 27-Gauge Short Needles Department of Pedodontics , Dental Faculty of Islamic Azad University of Khorasgan , Isfahan. JDMT 3:71–76

6. Hussain NUS, Younus S, Akhtar U Bin, et al (2020) COMPARISON OF PAIN PERCEIVED BY PATIENTS UNDERGOING INTRA ORAL LOCAL ANESTHESIA USING DIFFERENT NEEDLE GAUGES. Pak Armed forces Med J 70:1702–1706

7. Stuepp RT, Cabral Fenandes LG, Melo G, et al (2021) Anesthetic Efficacy of an Alternative Inferior Alveolar Nerve Block Technique Using an Extra-Short Needle: A Double-Blind Randomized Non-Inferiority Trial. J Oral Maxillofac Surg 79:1025.e1-1025.e8. https://doi.org/10.1016/j.joms.2020.12.038

8. Delgado-Molina E, Tamarit-Borrás M, Berini-Aytés L, Gay-Escoda C (2003) Evaluation and comparison of 2 needle models in terms of blood aspiration during truncal block of the inferior alveolar nerve. J Oral Maxillofac Surg 61:1011–1015. https://doi.org/10.1016/S0278-2391(03)00312-4

9. Mazhar B, Younus S, Akhtar U Bin, et al (2020) COMPARISON OF POSITIVE ASPIRATION DURING INFERIOR ALVEOLAR NERVE BLOCK USING CONVENTIONAL METALLIC DENTAL SYRINGE VS. DISPOSABLE PLASTIC SYRINGE. Pak Armed forces Med J 70:1539–1543
